# Malaria Elimination in Africa: Rethinking Strategies for *Plasmodium vivax* and Lessons from Botswana

**DOI:** 10.3390/tropicalmed8080392

**Published:** 2023-07-31

**Authors:** Isaac K. Quaye, Larysa Aleksenko, Giacomo M. Paganotti, Elias Peloewetse, Daniel H. Haiyambo, Davies Ntebela, Claude Oeuvray, Beatrice Greco, the PAVON Consortium

**Affiliations:** 1 Pan African Vivax and Ovale Network, Faculty of Engineering Computer and Allied Sciences, Regent University College of Science and Technology, #1 Regent Ave, McCarthy Hill, Mendskrom, Dansoman, Accra P.O. Box DS1636, Ghana; 2 Department of Health Sciences, School of Public Health, College of Health, Medicine and Life Sciences, Brunel University, Kingston Lane, Uxbridge, Middlesex, London UB8 3PH, UK; larysa.aleksenko@pavon.africa; 3 Botswana-University of Pennsylvania Partnership, Riverwalk, Gaborone P.O. Box 45498, Botswana; paganottig@bup.org.bw; 4 Division of Infectious Diseases, Perelman School of Medicine, University of Pennsylvania, Philadelphia, PA 19104, USA; 5 Department of Biological Sciences, Faculty of Sciences, University of Botswana, Gaborone Private Bag 00704, Botswana; peloewee@ub.ac.bw; 6 Department of Human, Biological and Translational Medical Sciences, Faculty of Health Sciences and Veterinary Medicine, University of Namibia School of Medicine, Hage Geingob Campus, Windhoek Private Bag 13301, Namibia; dhaiyambo@unam.na; 7 National Malaria Program, Ministry of Health, Gaborone Private Bag 0038, Botswana; dntebela834@gmail.com; 8 Global Health Institute of Merck, Terre Bonne Building Z0, Route de Crassier 1, Eysin, 1266 Geneva, Switzerland; claude.oeuvray@merckgroup.com (C.O.); beatrice.greco@merckgroup.com (B.G.); 9 PAVON, Regent University College of Science and Technology, #1 Regent Avenue, McCarthy Hiil, Mendskrom, Dansoman, Accra P.O. Box DS1636, Ghana

**Keywords:** *P. vivax*, Botswana, malaria elimination, *P. vivax* biology, malaria and sinusoids

## Abstract

The global malaria community has picked up the theme of malaria elimination in more than 90% of the world’s population in the next decade. Recent reports of *Plasmodium vivax* (*P. vivax*) in sub-Saharan Africa, including in Duffy-negative individuals, threaten the efforts aimed at achieving elimination. This is not only in view of strategies that are tailored only to *P. falciparum* elimination but also due to currently revealed biological characteristics of *P. vivax* concerning the relapse patterns of hypnozoites and conservation of large biomasses in cryptic sites in the bone marrow and spleen. A typical scenario was observed in Botswana between 2008 and 2018, which palpably projects how *P. vivax* could endanger malaria elimination efforts where the two parasites co-exist. The need for the global malaria community, national malaria programs (NMPs), funding agencies and relevant stakeholders to engage in a forum to discuss and recommend clear pathways for elimination of malaria, including *P. vivax*, in sub-Saharan Africa is warranted.

## 1. Introduction

The World Health Organization (WHO) Global Technical Strategy (GTS) for malaria is elimination by 2030 in 35 countries, while a target of global eradication by 2040 has recently been announced (https://www.who.int/publications/i/item/WHO-CDS-GMP-2019.10, accessed on 23 June 2023) [1]. The new affirmation follows from the earlier initiative of eradication launched in 2007 and the malERA initiative in 2011 [2,3]. The malERA elimination and eradication agenda has highlighted key challenges that need to be overcome to achieve elimination and eradication by the global community. These include (a) Prioritizing how to detect the foci of the burden of disease and drug resistance, and how they spread; (b) Ensuring a clear understanding of the operational process of surveillance and response based on scientific evidence; (c) Ensuring that the biology of *Plasmodium vivax (P. vivax)* is well understood and constitutes part of the strategies for elimination in all countries, including those in sub-Saharan Africa; (d) Sustaining capacity building and engagement efforts between scientists, policymakers and local actors so that the focus on elimination and eradication is clear and upheld [2,3,4].

The current strategy for malaria disease surveillance across sub-Saharan Africa involves using either Malaria Indicator Surveys (MIS), Demographic and Health Indicator Surveys (DHIS) or Multiple Indicator Cluster Surveys (MICS) as a sampling framework [5,6]. These surveys are modeled to obtain household information on malaria intervention coverage, prevalence of passive malaria cases, anemia and antimalaria drug use in pregnant women and children under 5 years of age. The surveys are managed centrally and conducted every 2–4 years because of the expenses involved, which makes them unrealistic to conduct more frequently. In addition, these passive surveys miss asymptomatic infections that silently increase and sustain transmission [5]. Additionally, they are not precisely standardized for detection. This approach is thus insufficient to provide information on disease burden and transmission, yet such information is needed to implement targeted interventions. While these problems exist in Africa, an additional barrier to elimination is the fact that African countries have held on to the perception that the burden of non-falciparum malaria is minimal, which has resulted in strategies for elimination being focused mainly on falciparum malaria. There is clear and established evidence of the presence of *P. vivax* and other non-falciparum (*P. malariae*, *P. ovale*) malaria in Africa [7,8,9]. In addition, *P. vivax* has been reported to cause severe malaria disease comparable to that caused by *P. falciparum* across all age groups [10]. It is, therefore, time for sub-Saharan Africa National Malaria Programs (NMPs) to rethink strategies for elimination to include *P. vivax* and other non-falciparum malaria. The good news is that for elimination, WHO recommends the implementation of active case detection (ACD) [11]. This recommendation makes it easier for policy implementers in Africa to carefully think through malaria elimination strategies that include non-falciparum malaria, particularly *P. vivax* [12]. The focus of the current article is to provide rethinking strategies based on current information on *P. vivax* biology and data on *P. vivax* transmission obtained over six years through active surveys in Botswana.

## 2. Life Cycle of Human *Plasmodium* Parasites and Their Recently Discovered Tissue Niches

### 2.1. Sporozoite Inoculation and Invasion of Tissues

The epidemiology of malaria depends on a complex interplay between the intermediate host, parasite, definitive vectors and the environment [13,14]. These factors differ from one geographic region to another, thus the epidemiology also differs accordingly. There are five *Plasmodium* species in the phylum *Apicomplexa* and order *Haemosporidae* that cause malaria in humans: *P. falciparum*, *P. vivax*, *P. ovale curtisi*, *P. ovale wallikeri*, (*P. ovale*), *P. malariae* and *P. knowlesi* [15]. An infection is initiated when a female *Anopheline* mosquito infected with the parasite inoculates an estimated 15–123 sporozoites under the skin of an individual while probing for a blood vessel to take a meal [16,17,18,19,20]. While under the skin, the sporozoites get activated based on the environmental milieu, express proteins required for gliding motility and cell traversal and travel through the blood to the liver using the sinusoids as entry points to hepatocytes [21,22,23,24]. The sinusoids are lined with resident macrophages, the Kupffer cells that aid in the transmigration of the sporozoites via gliding motility [25] through the sinusoids to reach hepatocytes [26,27]. Cell traversal proteins enable entry through the hepatocytes until one is selected for development; alternatively, the sporozoites may use the lymphatic system to enter tissues with the help of the sinus lining monocyte–macrophage system [28].

### 2.2. Entry into Extrahepatic Tissues/Cryptic Sites

**Figure 1:**
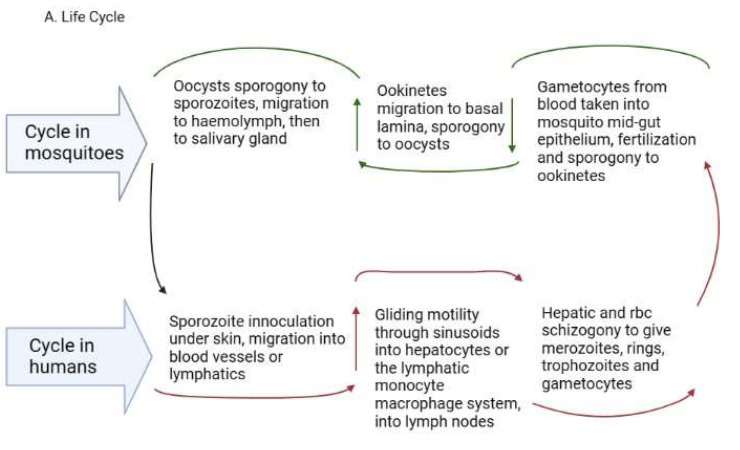

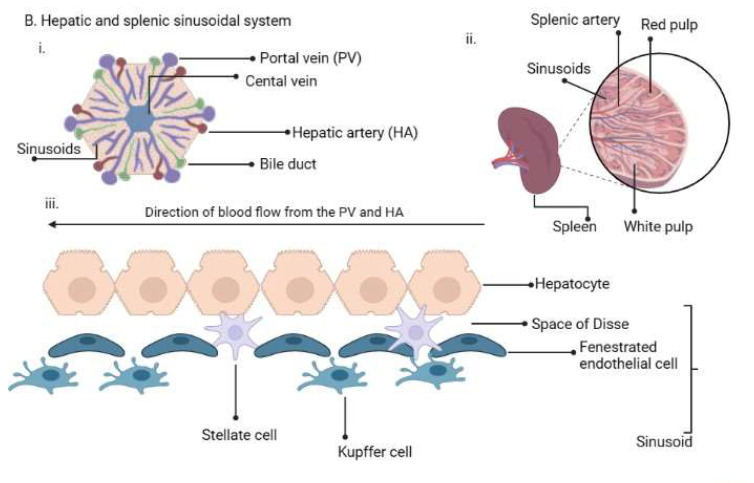
(**A**). *Plasmodium* life cycle. (**A**) Description of the *Plasmodium* life cycle from the sexual stage oocysts through sporogony to sporozoites, and migration into the salivary gland until inoculation into the human intermediary host during feeding. Sporozoites then migrate in the asexual stage into host tissues and development via schizogony and back to the sexual stages. (**B**) i. a liver lobule showing the triads, ii. a transverse section of the spleen showing an area of the sinusoid system, and iii. the hepatic sinus lining monocyte–macrophage system (sinusoid) Parasites seeking entry interact with the Kupffer cells and stellate cells through proteoglycans. Cells arrive at the sinusoids from blood flow coming through the intersection of the Portal vein and the Hepatic Artery in the liver. In the spleen, blood flows through the splenic artery into the central artery, either through the perifollicular zone and then through the venous sinusoids in the fast circulation, or the red pulp of the cords (consisting of fibroblasts and reticular fibers, but without endothelial cells) in a slow microcirculation. The slow circulation enables the mononuclear phagocyte system to remove particulate matter and generate the requisite immune response. From the red pulp, the blood squeezes through the venous sinusoids, which filter the blood further removing infected RBCs or intraerythrocytic bodies and exiting through the splenic veins to the portal vein.

It has recently been reported that sporozoites can access and develop in extrahepatic tissues of the body, including the spleen, bone marrow and lungs [17,29,30,31]. This raises the question of how the parasites enter these non-hepatic tissues or cryptic sites. In some vertebrates (birds and lizards), sporozoites can enter tissues and differentiate further using the monocyte–macrophage phagocytic system, including in the skin [25]. The Kupffer cells, which form the monocyte–macrophage system in the liver, are deficient in the production of reactive oxygen species (ROS), fail to respond to interferon-gamma during infection, are defective at secreting microbicidal molecules and serve as permissive hosts for intracellular parasites [32]. These characteristics have also been seen in spleen red pulp macrophages [33]. Comparatively, it may be that the culpable cells permitting intracellular parasite migration into non-hepatic tissues are the sinus lining monocytes/macrophages in the bone marrow, Langerhans cells in the skin, and alveolar macrophages in lung cells (Figure 1). These require further interrogation as they have a bearing on the role of relapses of hypnozoites in *P. vivax* and *P. ovale* malaria transmission [31] and asymptomatic infections. These could constitute new avenues for blocking malaria transmission using monoclonal antibodies.

### 2.3. Development of Asexual and Sexual Forms

The development within the tissues is impacted by the specific tissue environment. Rapid transcriptional changes occur in each parasite depending on the tissue milieu that determines the success of transmission [21]. In the liver, the sporozoites grow into liver schizonts that undergo schizogony and transform into merozoites, which are the red blood cell (RBC) infective forms [34]. The merozoites exit hepatocytes through the sinusoids and Kupffer cells to infect RBCs in the form of merosomes, which are merozoites enclosed in a membrane [35]. The merozoites use gliding motility similar to sporozoites for entry into RBCs [36].

In *P. vivax* and *P. ovale*, some sporozoites cease replicating and enter a dormant hypnozoite stage [37] from which they can relapse when triggered, entering a new replication mode and generating new merozoites [38]. Recent reports reveal that *P. vivax* hypnozoites express genes towards the gametocyte stage early in their development and seem programmed to commit to gametocytogenesis upon activation [39]. In this way, they serve as parasite reservoirs for propagation. In the RBCs, merozoites go through early ring forms, late trophozoites and blood schizonts that expand, burst through the RBCs and initiate a new cycle of infection as merozoites (Figure 1A). *P. vivax* merozoites are restricted to the invasion of reticulocytes while *P. falciparum* parasites are not restricted, although they show a preference for younger RBCs during invasion [40]. The schizonts contain about 16–32 merozoites [41]. The cycle of RBC invasion by merozoites leads to a paroxysm of fever that repeats every 24 h in *P. knowlesi*, 48 h in *P. falciparum*, *P. vivax* and *P. ovale* and 72 h in *P. malariae* [41]. These paroxysms give the respective descriptors of quotidian, tertian and quartian malaria, respectively, to the disease. During the asexual RBC cycle, some of the merozoites develop into immature male and female gametes called gametocytes, which mature to initiate the sexual cycle [42]. The immature forms, which are not visible in the peripheral circulation, are present in the bone marrow and spleen where they mature and re-enter the circulation [34]. In *P. falciparum*, five developmental forms have been observed and characterized as stages 1-V, although only the stage V form is seen in the peripheral circulation [43]. In *P. vivax,* however, all the asexual and sexual stages of the parasite can be seen in the peripheral circulation [44]. The mature gametocytes are taken up during feeding by anopheline mosquitoes and develop through gametocytogenesis to produce male microgametes and female macrogametes. The microgametes undergo exflagellation before fertilization with the macrogametes into zygotes [45] in the mosquito midgut (Figure 1A). Genetic recombination occurs during this period, leading to a diploid genome. The zygotes develop further into motile ookinetes in the midgut. Subsequently, the ookinetes move from the midgut epithelium to the basal lamina and develop into oocysts via a sporogonic cycle. Sporogony continues in the oocysts, generating numerous sporozoites [46,47]. Once mature, the sporozoites are released from the oocysts, after rupture, into the hemolymph and migrate into the salivary gland ready for a new cycle of inoculation and infection of humans [48] (Figure 1A). The genes expressed in the developmental cycle of the parasite appear to be species-specific based on the host and vectors that the parasite interacts with during the life cycle [48]. These differences are very important in the life cycles of *P. vivax* and *P. ovale*, which have hypnozoite stages. The gene expression pattern in the parasites is modulated based on host factors, allowing early release and viability of the gametocytes and sustaining transmission [44]. *P. vivax* is also established to adapt very well to climatic variations and multiple vectors, which adds to the difficulty associated with eliminating this parasite [47,49,50].

All the blood stages of *P. vivax* have been observed in the extrahepatic tissues (bone marrow and spleen). The immature gametocytes are enriched in the bone marrow parenchyma and the spleen [51,52,53], while the spleen is further enriched with late-stage asexual forms, trophozoites and schizonts [31]. Schizogony in the bone marrow and spleen can also generate merozoites for RBC infection [31]. Schizonts also accumulate in the lungs and adipose tissues [52,53]. These extrahepatic sites seem to be reservoirs for transmission and sources of sustenance of transmission, as they are protected from immune attack and drug treatment and occur in asymptomatic individuals [25,31,54]. Whether these synchronize with the liver stages to release merozoites into circulation is currently unknown.

### 2.4. The Reticuloendothelial (RES)/Mononuclear Phagocyte System (MPS) in the Plasmodium Life Cycle

The term ‘RES’ was originally coined by Karl Albert Ludwig Aschoff in 1924 [55] to reference cells involved in phagocytosis. The *Reticulo* referred to the tendency of these large cells to connect via cytoplasmic projections to form a network or reticulum, while *endothelial* referred to the closeness to the vascular endothelium. In later years, RES was renamed as cells of the mononuclear phagocyte system (MPS) [56] based on their morphology and kinetics and their ability to phagocytose. Currently, they are made up of three key cells based on their function and phenotype: monocytes, macrophages and dendritic cells [57]. The cells may be resident in their tissues or recruited, lining the sinusoids of their respective tissues. Macrophages are derived from embryonic progenitors, starting from the yolk sac and fetal monocytes and are subsequently distributed throughout the developing tissue through peripheral circulation [58,59]. These cells subsequently self-renew in the absence of adult hematopoiesis [60]. Monocytes, on the other hand, are derived from monocyte progenitor cells and dendritic cells from a dendritic cell precursor (adult hematopoietic stem cell precursors) [60]. Based on this classification, the previously classified RES cells—Kupffer, microglia, alveolar macrophages, splenic red pulp macrophages, Langerhans cells and lymphatic cells—are all considered as belonging to the macrophage type of cells as they are embryonically derived [60] (Figure 1B). An important point, though, is that while their origins are the same, the gene expression patterns are dependent on the tissues in which they are resident [61]. Interestingly, in all the indicated tissues, *Plasmodium* sexual and asexual parasites have been observed as discussed below, but much more studied in *P. vivax*.

### 2.5. RES/MPS as Host Sites of Cryptic Infections by Plasmodium Parasites

*Blood and Liver*: Sporozoites enter the bloodstream in the peripheral circulation using gliding motility [36], a process that is unique to *Apicomplexan* parasites. The parasites stay in the blood until they reach liver sinusoids, where they are initially sequestered. By interacting with heparan sulphate proteoglycans from Kupffer and stellate cells, they enter hepatocytes enclosed in a parasitophorous vacuole of host origin, via gliding motility, through the Kupffer cells [26,47,62] (Figure 1B). They finally settle in one hepatocyte to signal an end to the migration and continue to develop into schizonts. It has been suggested that cell entry within a parasitophorous vacuole limits cellular damage during migration, reduces the risk of an inflammatory response to the parasite and is used by the parasite for nutrient intake and efflux of waste materials [24,63]. Sinusoids present in tissues of the RES have been reported to harbor sexual and asexual forms of the *Plasmodium* parasites following human autopsy studies and rodent experiments [52,53]. A question that needs further interrogation is: do *Plasmodium* asexual and sexual forms have a preference for the use of sinusoids to enter tissues because of the peculiar role played by macrophages in sinusoid entry?

*Bone marrow and the spleen*: The bone marrow is a major site for erythropoiesis, while the spleen filters blood to remove old and damaged erythrocytes, particles and pathogens [64,65]. The spleen can contribute to erythropoiesis in a time of need, such as blood loss or trauma. The macrophages of the sinusoids and fenestrated endothelial cells play key roles during erythropoiesis and the filtering function of the spleen [66]. Bone marrow macrophages modulate erythropoiesis by close apposition to the developing erythroblasts, while during blood filtration in the spleen, macrophages recognize pathogens using pattern recognition receptors and pathogen-associated molecular patterns and remove them during the open and slow microcirculation through the sinusoids [67]. Parasite entry into the bone marrow and spleen is also suggested to be through gliding motility, like that of leukocytes during their transit to sites of acute inflammation, aided by tissue sentinel macrophages and molecular signals from the pathogens and damaged cells [68,69,70,71,72]. Therefore, a role for macrophages assisting in parasite entry, as mentioned previously, would be highly significant in the life cycle of the parasite. It has also been observed that merozoite-infected RBCs could home into the bone marrow and spleen in a receptor-mediated process due to vascular leakages that provide signals for invasion [53]. These observations were seen in both the rodent *P. berghei* parasite and the human *P. falciparum*, indicating that the bone marrow and spleen are major sites for parasite development during the *Plasmodium* life cycle. The unique biology of *P. vivax* and *P. ovale*, i.e., having hypnozoite stages, presents an even greater intriguing scenario considering that this stage can cause relapse after several years [73,74,75]. Although malaria relapses are attributed to a hypnozoite source in the liver, the current understanding of the life cycle may point to there being additional sources of cryptic ‘*non-liver rejuvenants*’ of dormant parasites [76]. A large proportion (>90%) of chronic *P. vivax* infections are asymptomatic, subpatent and submicroscopic [74]. These non-tangible infections have been observed to be present in the spleen and form the largest biomass of *P. vivax* infections, accounting for close to 98% of the biomass [31]. In addition, *P. vivax* genes involved in sequestration and cytoadherence are spleen-dependent [77,78]. Therefore, the spleen and bone marrow, in particular, serve as important sites in the life cycle of *P. vivax*. These cryptic infections must be considered carefully in surveillance strategies to ensure that tools for intervention are optimal. The use of passive case detection as currently conducted in most malaria-endemic countries in sub-Saharan Africa, focusing only on *P. falciparum* malaria, would fail to lead to malaria elimination.

*Lymphatic system*: The lymphatic system maintains tissue fluid homeostasis and coordinates the transport of immune cells to tissues [79]. The system harbors asexual *Plasmodium* parasites of all stages, including merozoites [18,80]. The source of the parasites could be sporozoites at the time of inoculation or directly from infected RBCs in the circulation [18], considering that the lymphatic system is closely linked with the hepatic sinusoids [81]. Parasite development could occur in the lymphatic system, leading to the generation of merozoites that can stay latent for some time before joining the peripheral circulation to invade new RBCs, hence sustaining the life cycle [80]. The subscapular macrophages of the lymph nodes may be the main cells permitting the entry of intracellular pathogens; this has been seen for viruses as well [82].

### 2.6. Plasmodium vivax Conundrum and Strategic Activities in an Infection

A pathogen’s ability to enable easy uptake by host phagocytes is described as the Trojan Horse model [83]. It appears that cells of the MPS are frequently used by intracellular parasites as Trojan Horses for entry into host tissues [84]. Prolonged environmental changes and interactions between pathogens and host cells are key drivers of adaptation and enablers of the spread or resilience during an infection [85,86]. These changes ensure some level of disengagement with or manipulation of the host immune system to ensure survival. The *Plasmodium* sporozoite has three main biological characteristics: to be motile, traverse cells and invade host cells [87]. For these functions, the sporozoites and merozoites have characteristic secretory granules: dense granules for modifying host cells, rhoptries for forming the parasitophorous vacuoles and micronemes for RBC invasion [88]. Sporozoites have been shown to contain rhoptries and micronemes, while merozoites have all three granules. Gliding motility is the main enabler of the three characteristics [88]. These characteristics are shared by *Plasmodium* parasites. These biological characteristics and cryptic infections will be discussed, taking into account malaria elimination and data obtained from Botswana from 2012 to 2018.

## 3. Trends of Malaria Transmission in Botswana from 2008 to 2018

The defined malaria elimination and eradication strategy is for countries to identify the foci of *Plasmodium* infections, treat the population and sustain surveillance to track any local transmission. From 2012 to 2018, we carried out systematic active studies of malaria transmission profiles in Botswana in collaboration with the National Malaria Program (NMP) to understand epidemiological outbreaks and provide data that could allow the NMP to target hotspots of infections covering all *Plasmodium* species. We present the findings together with the epidemiological outbreaks of malaria documented by the NMP from 2008 to 2012 and the predicted outlook for 2018. The findings from these studies reveal a profoundly complex transmission pattern of *P. vivax* and its interaction with *P. falciparum* for survival [89,90]. The observations are important and should be considered and factored into planning strategies for malaria elimination [14].

Botswana is a member of the Elimination 8 countries in Southern Africa and is one of the front-line countries that targeted malaria elimination in 2015 after concerted efforts to reduce the malaria disease burden to <1 per 1000 population by 2012 [91,92].

**Figure 2:**
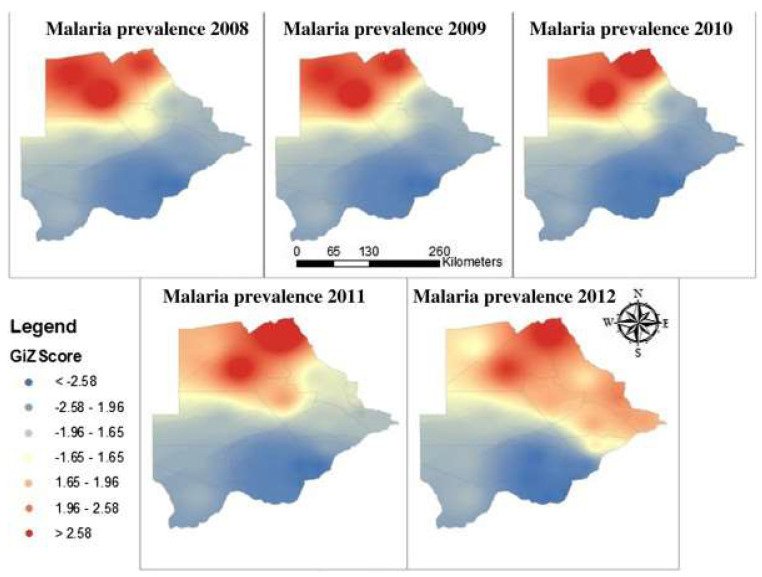
Annual patterns of malaria disease prevalence in Botswana from 2008 to 2012: The colors reflect the intensity of transmission from high (red) to low (deep blue) as indicated by the GiZ scores (*Source: NMP Botswana*).

These efforts included increased awareness campaigns towards the uptake of the use of long-lasting insecticide-treated nets (LLINs), indoor residual spraying (IRS), increased use of rapid diagnostic tests (RDTs) for diagnosis [93] and introduction of artemisinin combination therapy (ACT) for treatment. Unfortunately, the projected target for elimination could not be achieved because of challenges in detecting asymptomatic infections and sporadic cases of malaria localized in the north, eastern, southeastern and parts of the southern regions [93] (Figure 2). The failure was partly because the surveillance strategy was based on passive case detection, which did not account for asymptomatic infections and non-falciparum malaria [94]. The burden of malaria disease has traditionally been in the northern part of the country, which is wetter and warmer compared to the southern part of the country. Occasionally, the relatively wetter parts in the eastern, southeastern and southern parts of the country also experience sporadic transmission, depending on the rainfall patterns [93]. Rainfall is seasonal and occurs between November and May [93,95], thus the transmission season occurs over the same period. *Anopheles arabiensis* (*An. arabiensis*) accounts for most of the malaria transmission in Botswana, seen in all districts with noted malaria transmission [96]. Within the *An. gambiae* complex, it is described as partially zoophilic, exophagic and exophilic. The *P. falciparum* parasite rate of the vector in Botswana can be up to 3% in pyrethrum spray catch collections, depending on the region, time of year, annual rainfall and human parasite reservoir [97]. It is known to transmit both *P. falciparum* and *P. vivax* in Ethiopia [98]. Surveys in Botswana and surrounding countries have also revealed the presence of *An. funestus* s.l., the second most important taxon of malaria vectors in Africa [96,97,99]. Within the *An. funestus* group, a study in 2017 confirmed the presence of *An. parensis* in Okavango, Ngami, Chobe and Bobirwa districts, while *An. longipalpis type C* was found in Ngami and Boteti districts [97]. *An. quadriannulatus,* which is predominantly zoophilic, has been found in Ngami, Chobe, Bobirwa and Kweneng West districts [97]. *An. funestus* transmits all four *Plasmodium* species in Kenya [100]. The roles of other *Anopheles* species in malaria transmission in Botswana have not been well documented [96,99].

### 3.1. Recorded Annual Trends

**Figure 3:**
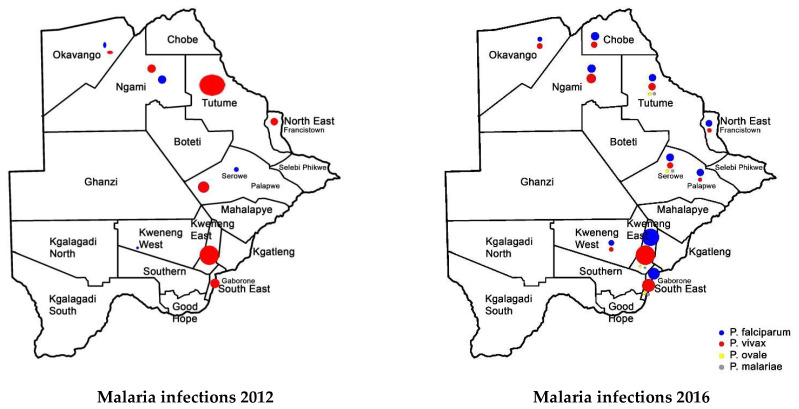
Malaria infections in 2012 and 2016–2017. The sizes of the circles represent *Plasmodium* parasite prevalence at the sites.

**Figure 4:**
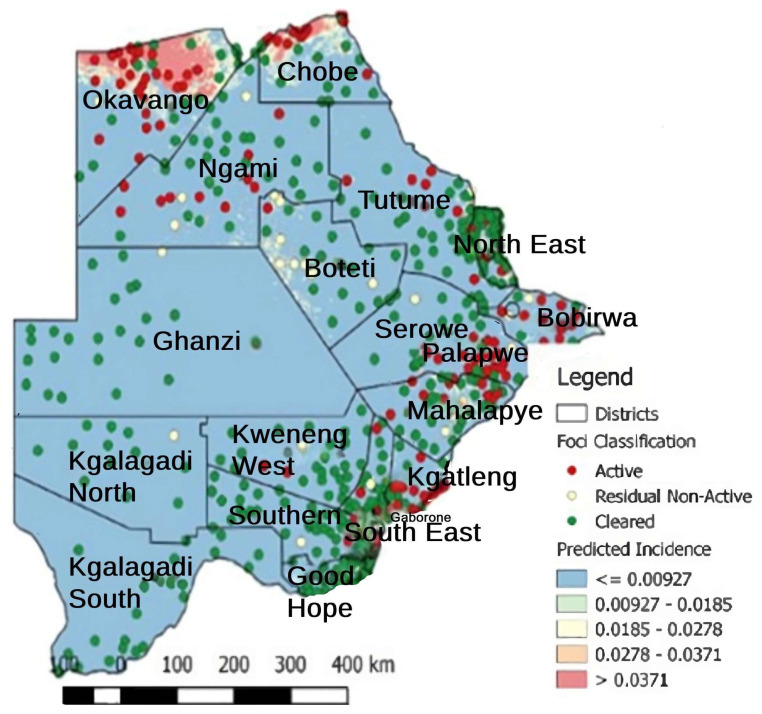
Predicted malaria prevalence in 2018. The red circles indicate active expected incidences in the respective areas, while the green circles indicate areas expected to be free from malaria. The size of the circles is only an indicator and does not reflect intensity. The color on the map reflects transmission: red (high) and blue (low). *Source: NMP Botswana*.

In 2008 and 2009, the foci of malaria disease burden were in three districts: Okavango, Chobe, and Ngami in the north (Figure 2). By 2010, the burden in Okavango had diminished marginally, with the foci remaining localized in Ngami and Chobe (Figure 2). At the same time, a small node of transmission emerged, running from Ngami towards Boteti; this became significant in 2011 (Figure 2). By 2012, the entire geographical area from the northeastern section towards the southeastern border regions was experiencing significant and sporadic malaria outbreaks. These outbreaks were seen despite a general reduction in malaria disease burden to <1 per 1000 populations, with reference to *P. falciparum* as the main causative parasite. It was a puzzling occurrence and after several discussions on the way forward, it was decided that an active survey was needed to unravel some elements of the puzzle. In 2012, and considering that the target for malaria elimination was 2015, an active survey was conducted across the country within the transmission period and species-specific nested PCR was used for detection of all *Plasmodium* species and asymptomatic infections. The result of this survey was profound, as previously published [89]. We confirmed that *P. falciparum* was mainly localized in the Kavango and Ngami regions (no samples were collected in Chobe at the time), with some asymptomatic infections. However, we observed, for the first time, that asymptomatic *P. vivax* infections were present and localized at hotspots across the country [89]. Areas where the asymptomatic *P. vivax* burden was found mirrored the observed outbreaks in a way that suggested that they were part of the recorded epidemic outbreaks. In addition, areas in the South (Kweneng) that had experienced occasional outbreaks but were deemed to be unimportant (Figure 2) were identified as major foci of asymptomatic *P. vivax* infections. These findings corroborated the observed characteristics of *P. vivax* transmission, which is that asymptomatic *P. vivax* infections contribute to significant transmission and account for the majority of transmissions in low-endemic settings, as observed elsewhere [101,102]. In addition, they were highest in areas where *P. falciparum* was not detected by nested PCR, indicating a reciprocal interaction between the two parasites, as has been generally observed. Since samples were collected during the transmission season, we could not claim relapsing *P. vivax* hypnozoites. Two questions were on our minds: if these were partly hypnozoites that relapsed, (a) what triggered the activation and (b) were these relapses from liver hypnozoites only? Since the outbreaks were spread out over a large area but within the same period, we wondered whether the trigger/triggers was/were something in the environment that led to a physiological response and activation or whether it was due to an innate biological clock that spontaneously induced an activation. We also wondered whether parasite responses to a trigger were spontaneous within a given geographical area (what we would term a ‘*Spoke wheel effect*’) (Figure 3) or ‘*relayed*’. Following the extension of the elimination agenda to 2018, to gain insights into the nature of the observed *P. vivax* parasites, in 2016 we embarked on another survey at the same sites but included Chobe, which we had previously missed. This time we collected samples all year round, from August 2016 to October 2017, and added qPCR to increase the sensitivity of detection. The findings were particularly interesting. We noted that not only was *P. vivax* still present in the sites where they were previously identified, but for some unknown reasons, *P. falciparum* had re-appeared in all the places where *P. vivax* had been observed. In addition, with the return of *P. falciparum*, the burden of *P. vivax* diminished but did not disappear, allowing for coexistence within the communities. Interestingly, we identified *P. vivax* in the non-transmission season, indicating a role in the relapse of hypnozoites in the population. The active survey reflected the epidemic outbreak in the 2016/2017 period, showing an 80% increase in malaria transmission in that period [96,103]. In 2018, the NMP mapped areas of active malaria transmission across the country using a modeling approach (Figure 4). It was evident that the predicted active sites were consistently in areas where asymptomatic *P. vivax* infections had been detected. Not surprisingly, the elimination date was revised to 2025 following a 2021 review by the Malaria Elimination Oversight Committee (MEOC) of the WHO to include Botswana in the Elimination 2025 (E-2025) countries [104].

### 3.2. Malaria Elimination Successes in the WHO Regions of Africa and the Americas

A key question that naturally comes to mind is what strategies would be required to ensure successful malaria elimination in a low endemic setting such as Botswana, taking lessons from countries within the E-2020 group that succeeded and where *P. vivax* co-existed with *P. falciparum*. Two regional examples will be briefly examined here for lessons: Algeria in the WHO Africa Region and Belize and El Salvador in the WHO Region of the Americas. The key lessons that were gleaned from the success in Algeria, which is the country where the *Plasmodium* parasite was detected by Alphonse Laveran in 1880 [105] were: a well-trained malaria disease workforce, rapid response to disease outbreaks, political commitment to the provision of domestic funding to ensure effective case-based surveillance, robust data management systems, easy access of the community to care and community engagement [106]. These fundamental principles and activities were owned and sustained continually until elimination. El Salvador had the highest cases of malaria in Central America in 1965 but was certified malaria-free in 2021; the first country in Central America to eliminate malaria [106]. Belize has followed suit in Central America and was certified malaria-free in early 2023. The countries used approaches similar to those enumerated for Algeria, based on WHO-prescribed procedures and support, while also adding local initiatives in innovation that fitted their local conditions to facilitate the elimination process. We think that part of the innovative activities would be coupling NMP surveillance of all *Plasmodium* parasites and asymptomatic infections with diagnostics and detection, disease mapping and modeling by institutions that have established strengths in these areas.

## 4. Discussion

We have presented the pattern of malaria disease outbreaks in Botswana and their congruence with the foci of *P. vivax* infections across the country [87,88]. This pattern has not been seen previously, raising the need for critical thinking about trends of malaria disease outbreaks in Botswana and areas with low endemicity in Africa, taking into consideration the probable role of *P. vivax*. We have shown here that an active malaria survey under low transmission or elimination agenda is a powerful tool that can presage epidemic outbreaks. Of all the surveys presented here, asymptomatic parasites detected using the active surveys precisely mirrored the epidemic outbreaks that followed. This confirmed previous observations that asymptomatic *P. vivax* infections contribute to malaria disease transmission [74,107,108,109]. We also observed, for the first time, that asymptomatic *P. vivax* infections can drive the re-emergence of *P. falciparum* in an area; however, the mechanism through which this occurs is currently unknown. It is clear that once established in an area, *P. vivax* parasites become entrenched and require a vigorous test-and-treat approach using anti-blood-stage and tissue-stage drugs to eliminate them. A *P. vivax* strategy using a serodiagnostic tool, as proposed recently, will be needed in the elimination agenda [110]. Considering that all African countries that have reported the presence of *P. vivax* have also reported the presence of infected Duffy-negative individuals, the road to malaria elimination in Africa will not be easy [7] and will demand a rethinking of strategies that consider all malaria species as targets for elimination as a must-do activity [12]. The success stories from previously mentioned WHO regional countries provide primary leads for NMPs, including in Botswana, to follow. These include innovative activities that are locally tailored to ensure that the goal of malaria elimination is achieved.

The new biological characteristics identified in the *P. vivax* life cycle with cryptic infections detected in extrahepatic sites (spleen, bone marrow and the lymphatic system) present a new conundrum. It is of interest that in all the cryptic sites, the sinusoidal system is used by the parasites to enter tissues. The sinusoids have a unique anatomical structure that includes a monocyte–macrophage system in addition to the fenestrated endothelium [35]. All the tissues are therefore involved in defending the body, particularly the spleen, where the largest biomass of *P. vivax* infection has been found. Could it be that the Trojan Horse model approach described previously is a parasite strategy of infection that has not been duly considered? [83]. If the parasites adapt successfully to using the defense tools in tissues as a way of escape, then effective countermeasures such as anti-blood-stage and tissue-stage drugs that are not restricted—as is the case with the 8-aminoquinolines in G6PD deficiency—are needed. *Leishmania donovani* and related species that cause visceral leishmaniasis are known to spread throughout the body using the mononuclear phagocyte system [111,112]. Growth in the MPS is vigorous in the case of these parasites, leading to the destruction of tissues and their dissemination in the body. In the spleen, splenomegaly is seen before tissue destruction [113]. The initial immune response to an infection in mice is TH1-dependent, which subsides from TH2 responses to reduce inflammation but leads to a chronic infection and tissue destruction [114,115]. It appears the TH1/TH2 balance fails in humans such that growth continues vigorously, leading to death [116]. It is interesting that the two tissues in which high numbers of *P. vivax* parasite biomass have been observed (spleen and bone marrow) are also hematopoietic. When one considers that *P. vivax* is restricted to using reticulocytes, it may be that the parasites modulate the hematopoietic tissues to increase erythropoiesis to enable them to thrive. This mechanism of infection has been demonstrated in *L. donovani* infections in hamsters [117]. The spleen is known to supplement hematopoiesis under trauma and stress [66]. In a study conducted in Colombia, under low endemic settings where *P. vivax* and *P. falciparum* exist, all patients with uncomplicated malaria who had hepato-splenomegaly were infected with *P. vivax* [118]. The frequency of pallor and hemoglobinuria were higher in *P. vivax* than in *P. falciparum* infections [118]. These clinical signals in uncomplicated *P. vivax* malaria should be documented in clinical presentations to facilitate the diagnosis of *P. vivax* infections in low-endemic settings. The mechanism of infection of extra-hepatic tissues by *P. vivax* parasites warrants thorough investigations.

## 5. Conclusions

We have shown that the presence of *P. vivax* appears entrenched in low transmission environments in Botswana. This could be attributed to the complex biology of the parasite in the human host. The global health community needs to have a discourse on this and provide guidelines for NMPs to follow. Without such input for African NMPs, the path to elimination will be formidable. This engagement should also consider available diagnostic options, active case detection and treatment with 8-aminoquinolines. Bottlenecks and challenges that program implementors face to put WHO drug recommendations for the radical cure of *P. vivax* infections into practice must also be assessed and recommendations provided.

## Data Availability

Not applicable.
